# Prebiotic Lipidic Amphiphiles and Condensing Agents on the Early Earth

**DOI:** 10.3390/life6020017

**Published:** 2016-03-28

**Authors:** Michele Fiore, Peter Strazewski

**Affiliations:** Institut de Chimie et Biochimie Moléculaires et Supramoléculaires (Unité Mixte de Recherche 5246), Université de Lyon, Claude Bernard Lyon 1, 43 bvd du 11 Novembre 1918, 69622 Villeurbanne Cedex, France

**Keywords:** prebiotic chemistry, hydrothermal conditions, origin of life, amphiphiles, lipids, phosphorylation, phosphite, cyanamide, urea, vesicles

## Abstract

It is still uncertain how the first minimal cellular systems evolved to the complexity required for life to begin, but it is obvious that the role of amphiphilic compounds in the origin of life is one of huge relevance. Over the last four decades a number of studies have demonstrated how amphiphilic molecules can be synthesized under plausibly prebiotic conditions. The majority of these experiments also gave evidence for the ability of so formed amphiphiles to assemble in closed membranes of vesicles that, in principle, could have compartmented first biological processes on early Earth, including the emergence of self-replicating systems. For a competitive selection of the best performing molecular replicators to become operative, some kind of bounded units capable of harboring them are indispensable. Without the competition between dynamic populations of different compartments, life itself could not be distinguished from an otherwise disparate array or network of molecular interactions. In this review, we describe experiments that demonstrate how different prebiotically-available building blocks can become precursors of phospholipids that form vesicles. We discuss the experimental conditions that resemble plausibly those of the early Earth (or elsewhere) and consider the analytical methods that were used to characterize synthetic products. Two brief sections focus on phosphorylating agents, catalysts and coupling agents with particular attention given to their geochemical context. In Section 5, we describe how condensing agents such as cyanamide and urea can promote the abiotic synthesis of phospholipids. We conclude the review by reflecting on future studies of phospholipid compartments, particularly, on evolvable chemical systems that include giant vesicles composed of different lipidic amphiphiles.

## 1. Introduction

Phospholipids belong to the most important cellular constituents. Their biosynthesis is well understood [1]. Lipids, or lipid-like amphiphilic structures, played a crucial role in the early stage of our planet in creating the necessary boundary structures capable of encapsulating and harboring other biomolecules [2]. Such amphiphiles thus generated the minimal autopoietic structures that gave birth to the cell’s cenancestor [3]. The boundaries consisted of closed yet semipermeable compartments that enwrapped the essential components required for replication, translation, and transcription [4].

What lipid-like compounds might have been available to assemble into primitive membranous boundaries? Organic oxygenated compounds such as phenols and carboxylic acids have been identified in carbonaceous meteorites [5,6,7], and some components of the extracts were capable of forming membranous compartments upon hydration at alkaline pH ranges [5]. Lipids have also been proposed as ideal biomarkers for samples of extraterrestrial material [8]. However, like carbohydrates, and unlike certain phenols, N-heterocycles, alkanes, carboxylic acids, and amino acids, lipids are chemically and thermally relatively labile over geological timescales. Extracts from the remnants of extraterrestrial objects that entered the Earth’s atmosphere (meteorites), or from samples taken by a lander instrument (on planets, moons, asteroids, and comets) are expected to contain at best degradation products of lipids, *viz.* alkanes, long chain alcohols, polyols, and carboxylic acids. It is yet to be determined how the different lipid precursors formed abiotically to give more complex molecules like phospholipids [9]. Several proposals offer plausible scenarios for the formation of amphiphilic structures related to life’s origin [8,9,10,11,12] and many experiments have been carried out to investigate the synthesis of amphiphilic material under plausibly prebiotic conditions. In most of the cases, the products were first analyzed and chemically characterized, then used for the preparation of vesicles. The design of these experiments took advantage of the pioneering Miller-Urey experiments on the abiotic formation of amino acids and hydroxy acids [13].

Hence, in view of the inherent natural and technical difficulties in obtaining convincing geological or exobiotic evidence on the true sequence of historical events that led to life on Earth, a smart alternative was “to reinvent” prebiotic chemistry through experimental simulation in the laboratory, in order to better understand the chemical origin of amphiphiles and other vital molecules [14]. Over the past 50 years or so, dozens of chemists and a few synthetic biologists followed this strategy. We shall summarize the “state of the art” of prebiotic synthesis of phospholipids under prebiotic conditions.

## 2. Geochemical and Historical Contexts

Microfossil evidence indicates that cellular life on Earth emerged during the Paleoarchean era between 3.6 and 3.2 thousand million years ago (Gya) [15,16]. Chang [17], Oró [18], Lazcano [19], Pace [20], Eschenmoser & Loewenthal [21], Orgel [22], Bada [23], Snooks [24], Root-Bernstein [25] and many others reviewed the chemical and biological constraints on life’s origin on Earth, including the inherent philosophical implications. Kuhn [26], Pross [27,28], Pascal & Pross [29], and Luisi [30] among others, highlighted the problems of understanding the transition from chemical to biological processes and the abiotic synthesis of biomolecules. The goal now is to understand, perhaps from experimental reproduction [31], the formation of a chemical system that is self-sustained, kinetically stable, dynamically evolvable, and far from thermodynamic equilibrium, which we then could call “alive” or “animate”.

To date, because it is impossible to separate historical from ahistorical events, it is necessary to clarify what is plausible and then combine experimental and observational evidence to obtain convincing answers. It is probable that the prebiotic formation of the first membrane-forming amphiphiles occurred in the Eoarchean era 4.0–3.6 Gya and coincided with the appearance of prebiotic amino acids and N-heterocycles (to become nucleobases), all produced from then available geochemical sources, including extra-terrestrial material [32]. At that time, sunlight, volcanic heat, and hydrothermal sites were the main energy sources that could drive the synthesis of amphiphilic compounds. A crucial feature was fluctuations of hydrated and dehydrated conditions related to precipitation and evaporation of water on volcanic land masses [33]. The atmosphere was mostly nitrogen (N_2_), as today, with a substantial amount of carbon dioxide (CO_2_) and much smaller amounts of carbon monoxide, ammonia, and methane (CO, NH_3_, CH_4_). It is also likely that water, present in locally limited amounts, contained hydrogen cyanide (HCN), formaldehyde (HCHO), and formamide (HCONH_2_) [34,35]. Different geological events allowed for prebiotic reactions that led to the synthesis of biologically significant building blocks [36]. Eschenmoser & Loewenthal formulated in 1992 for the first time a rationale on a comprehensive “chemical etiology” of a number of biotically relevant N-heterocycles, ultimately all originating from HCN, and published the pioneering “hydrocyanic origin” of purines and cofactors considered necessary for life’s origin [21]. Recently, Sutherland proposed that RNA, certain amino acids, and lipids may bear common ancestral building blocks. Sutherland & co-worker’s research of the last years demonstrated that these building blocks could be prebiotically formed from the reductive homologation of HCN, where hydrogen sulfide (H_2_S) and bright light provide the reaction mixtures with reducing power. A hypothetical systems chemistry cascade (reaction network) was outlined and termed “cyanosulfidic proto-metabolism” [37,38]. The future will tell whether autocatalytic, crosscatalytic or collectively autocatalytic feedback loops can be identified, which would render this cascade a sustainable reaction network, so that free energy could be harvested from it.

## 3. Classification of Prebiotic Amphiphilic Material as “Incomplete” and “Complete” Lipids

### 3.1. Abiotic Retrosynthetic Analysis

**Figure 1:**
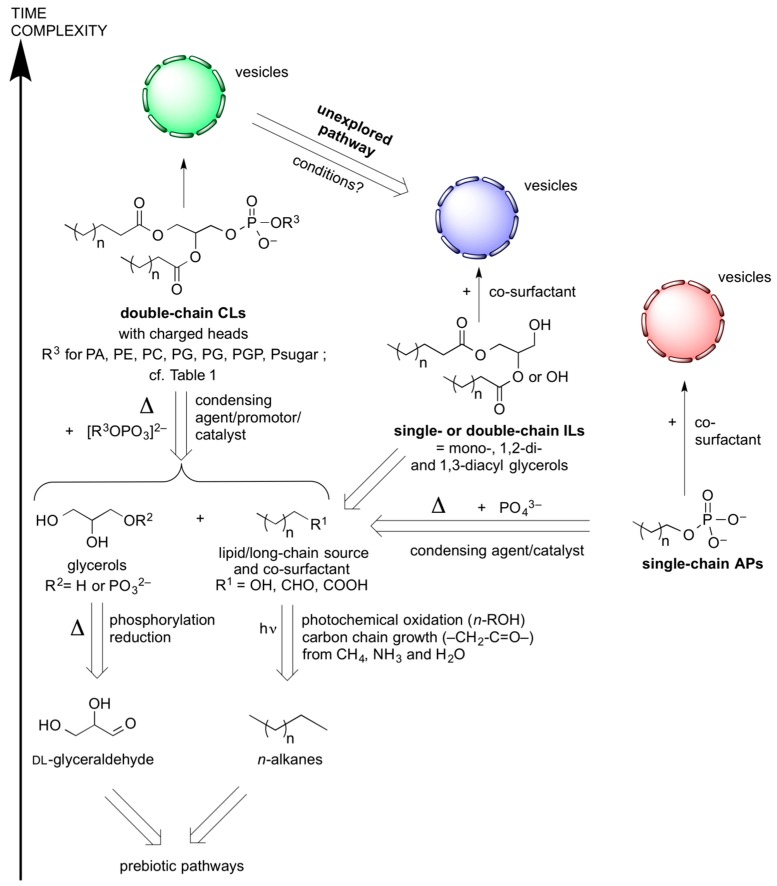
Possible abiotic retrosyntheses (indicated by open arrows) of long-chain alkyl phosphates (APs), single- and double-chain “incomplete” lipids (ILs) and “complete” lipids (CLs). The pathways are referring to the plausibly prebiotic reaction conditions that are listed in Table 2 and Table 3. Most of the extracted amphiphilic material was able to form liposomes or similar vesicular supramolecular structures; this is schematically represented at the top of the figure by colored vesicles bearing (symbolically) semi-permeable membranes.

We can now define parameters for the most plausible synthesis of lipids (long-chain diacyl-glycerol derivatives) and phospholipids (long-chain diacylglycerol phosphate derivatives) under prebiotic conditions. We shall define two classes of lipids: we will refer to those lacking a polar phosphate headgroup as “incomplete lipids” (ILs), and those that have a phosphate will be called “complete lipids” (CLs). Figure 1 summarizes the abiotic retrosynthesis of ILs and CLs. An implicit assumption behind this analysis is that prebiotically formed amphiphiles (“pre-Darwinian” amphiphiles, before protocellular replication set in), which assemble into membranes and close into semi-permeable boundaries of vesicular compartments (with a void volume inside), must be racemic (if chiral). Prebiotic membrane forming amphiphiles are most likely acids and esters or perhaps amides, rather than ethers, as found in Archeal phospholipids [9], or long-chain alkenyl compounds such as present in ceramides and sphingolipids. The reaction conditions needed to form carboxylic and phosphoric esters (or amides) from the corresponding acids and alcohols (or amines) are considered feasible, because a mild prebiotically plausible activation in water often suffices. The formation of carbon-oxygen bonds to obtain ethers from two alcohols requires much harsher reaction conditions, and leaving groups that were too prone to hydrolyze or eliminate instead.

In anticipation of our final remarks (Subsection 6.2) we are emphasizing in this figure that a sufficient integrity of vesicular structures is required in order not to interrupt the co-evolution of their entrapped macromolecular contents. The simulated prebiotic formation of lipidic amphiphiles thus also includes single-chain lipids and long-chain alkyl phosphates (APs). To form vesicles, both ILs and APs require significantly higher (critical vesicle) concentrations than CLs bearing the same alkyl chain lengths. The unphosphorylated variants of those simpler amphiphiles, however, are known to promote and stabilize vesicle assembly as “co-surfactants”, which may help evolve the complexity of the chemical composition of the vesicles without significant leakage of their macro-molecular contents. The plausibly prebiotic synthesis of ILs, CLs, and APs is treated separately in Subsection 3.2 and Subsection 3.3. In most of the reported simulations, it is clear that three chemically distinct starting ingredients were prerequisites: (a) a source of long-chain “fatty” acids, aldehydes, or alcohols, (b) a polyol scaffold like glycerol that can bear one or two lipophilic chains and (c) a source of phosphate such as inorganic orthophosphate PO_4_^3*−*^, or R^3^OPO_3_^2*−*^ like glycerophosphate, for the direct synthesis of CLs. In Table 1, we list the different types of CL’s polar headgroups that may be formed under plausibly prebiotic conditions.

### 3.2. Prebiotic Formation of ILs

Considering UV radiation as a plausible source of energy on prebiotic Earth, Klein and Pilpel prepared *n*-decanoic and *n*-hexadecanoic alcohols by photochemical/radical oxidation of the corresponding *n*-alkanes (*n*-RH) by using 1-naphthol for photoactivation [39]. The reaction proceeded from *n*-alkane radicals that then were oxidized to peroxide radicals. These formed hydroperoxides (*n*-ROOH) that reacted with another *n*-RH molecule to give, in an overall conproportion process, two molecules of long-chain alcohols (*n*-ROH). The conversion to peroxides was poor while the conversion into *n*-ROH reached 90%. The compounds were characterized by GC-MS, IR, and NMR spectroscopy. Remarkably, those *n*-ROOH (then *n*-ROH) that formed from alkanes bearing even numbers of carbon atoms were more abundant than those that formed from alkanes bearing odd numbers of carbon atoms, and the stability of *n*-ROOH increased with the chain length. A few years later, by simulating presumed Archean atmospheric conditions [40], Simionescu and co-workers showed that cold condensed gas mixtures of CH_4_, NH_3_, and H_2_O produced ill-defined aldo- and keto- compounds of medium molecular weight (*m/z* 128 to 559). The presence of keto-groups –CH_2_–C(=O)– was elucidated by ^13^C-NMR spectroscopy. These mixtures of “hydrophobic” molecules were capable of self-assembling into supramolecular liposome-like structures very similar to vesicular lipid microspheres [41]. Similar results were obtained by McCollom and co-workers who optimized a Fischer-Tropsch type reaction from the disproportion of ^13^C labelled formic acid or oxalic acid under simulated hydrothermal conditions (175 °C, days). They identified C_2_ to C_35_ *n*-alkanols, *n*-alkanoic acids, *n*-alkenes, *n*-alkanes and alkanones [42]. The same authors also demonstrated that amphiphilic oxygenated compounds bearing the empirical molecular formula “C_n_H_2n–6_O” and medium molecular weights (*m*/*z* 258 to 262) were formed by thermal decomposition of siderite, *i.e.*, iron oxalate dihydrate (FeC_2_O_4_**^.^**2 H_2_O) at 400 °C [42]. More complex structures were obtained when glycerol and “fatty” (long-chain) aliphatic chains were condensed in the presence of a catalyst or a coupling agent under simulated prebiotic conditions.

At this point, it is important to distinguish between conditions that are described as “hydrothermal”. A “hydrothermal synthesis” usually refers to heterogeneous reaction conditions in aqueous media at 100–400 °C and 1 bar, such as the preparation of synthetic minerals [43], whereas so called “simulated hydrothermal conditions” or “hydrothermal prebiotic conditions” refer to reactions that are favorable for the abiotic synthesis of biomolecules [44]. In this section, we compiled the experiments that have been carried out under “simulated prebiotic conditions” (65–100 °C) and “simulated hydrothermal conditions” (150–300 °C) to produce different ILs, either bearing a single chain or more complex structures, like mono-, 1,2- and 1,3-diacyl glycerols. Thus, Oró and co-workers obtained mono-, 1,2-di-, 1,3-di- and tripalmitoyl glycerols from the condensation of ammonium palmitate with glycerol after heating (100 °C) for relatively short periods of time (hours) under different conditions and in reasonable yields [45]. The crude products were separated by TLC, analyzed by ^14^C-autoradiography (^14^C-glycerol was used for labelling the products) followed by the enzymatic transformation of the formed products to confirm the presence of those compounds in the extracted mixtures. Notably, for the first time, a condensing agent/catalyst mixture, such as cyanamide/imidazole, was used (Table 2, Entry 5). Later, similar results for similar products and yields were obtained by Rushdi and Simoneit [46] and Simoneit and co-workers [47] by heating under “simulated hydrothermal conditions” *n*-nonadecanoic acid and *n*-heptanoic acid and glycerol in the presence or absence of oxalic acid yielding 1,2- and 1,3-diacyl glycerols as condensation products, which were characterized by GC-MS. Organic extracts of the mixtures self-assembled in vesicular structures. In Table 2, entries 1–6, we summarize the most relevant simulations of prebiotic syntheses of amphiphilic lipidic material under different chemical conditions.

### 3.3. Prebiotic Formation of CLs and APs: Role of the Phosphorylating Agent

The first experiments on the synthesis of CLs like phosphatidic acids (PA) began in the late 1970s. Deamer and co-workers reported that ^14^C-glycerol, *n*-dodecylic acid (or aldehyde) in the presence of sodium dicyanamide and disodium hydrogen phosphate (Na_2_HPO_4_) yielded PA, PG (phosphatidylglycerol), and PGP (phosphatidyl glycerophosphate) in low amounts (0.02%–0.2%). The compounds were characterized after purification by TLC and ^14^C autoradiography, and by comparison with original phospholipid samples. For the first time, crude extracts of those materials, once hydrated, were reported to yield vesicular supramolecular assemblies [48]. In the same years, Oró and co-workers reported on the synthesis of MPGP (monopalmitoyl glycerophosphate), DPGP (dipalmitoyl glycerophosphate), and cMPGP (cyclic monopalmitoyl glycerophosphate) by condensing ^14^C-glycerol-1-phosphate with ammonium palmitate in the presence of cyanamide [49]. Notably, the same authors, by reduction of dl-glyceraldehyde, obtained glycerol that was phosphorylated using ammonium dihydrogen phosphate, NH_4_(H_2_PO_4_) [50]. Phosphatidylcholine (PC) synthesis was achieved by condensing choline chloride in the presence of Na_2_HPO_4_ with optimized yields (15%) at 80 °C [51]. In 2014, Pasek and co-workers repeated these experiments, and characterized, among others, phosphate esters obtained by direct condensation of glycerol and phosphate in “deep eutectic solvents” (DES) defined as 2:1 mixtures of urea and choline chloride [52]. Relevant phosphate esters like *sn*-glycerol-3-phosphate (PGP), glycerol-2-phosphate and phosphoethanolamine (PE) were synthesized in low yields under “hydrothermal conditions” by using different phosphate sources and minerals as catalysts [53]. More recently, taking inspiration from the remarkable supposition that early vesicle bilayers may have formed from single chain amphiphilic material [54], Monnard and Sutherland reported that the condensation of *n*-decanol at 100 °C in the presence of urea and ammonium dihydrogen phosphate NH_4_(H_2_PO_4_) gave *n*-decyl phosphate [55]. Hydrated organic extracts of the crude material produced vesicles in the presence of different co-surfactants. The compounds, after ESI-MS analysis, were identified through the comparison with synthetic samples [56]. Despite being not strictly prebiotic, it is interesting to report that dialkylphosphates were also synthesized by reacting *n*-decyl phosphate with alkene oxides [57]. In Table 3, entries 7–14, the conditions for the synthesis of CLs and APs under plausible prebiotic conditions are summarized.

## 4. Availability and Role of Phosphorylating Agents in the Formation of Phosphorous Containing Amphiphiles

### 4.1. Condensed Phosphates

All simulated prebiotic phosphorylation experiments leading to the formation of APs and CLs with (Table 3, entries 8 and 10–14), combined orthophosphate P_i_ as sodium or ammonium salts with anhydride condensing agents, a strongly dehydrating milieu like DES, elevated temperatures or combinations thereof. In 1988, Morowitz, Heinz, and Deamer [58] proposed a hypothetical alternative, according to which the phosphorylating and condensing action could be driven by pyrophosphate (P_2_O_7_^4−^ = PP_i_). Long-chain alcohols *n*-ROH might be transformed into APs (or CLs) and orthophosphate (PO_4_^3−^ = P_i_) according to Equation (1).

*n*-ROH + PP_i_ → *n*-ROPO_3_^2−^ + P_i_ (1)

Although there is, to our knowledge, no experimental evidence for this, an intervention of PP_i_ in the formation of APs, or CLs, starting from polyols (sugars, glycerol) and a source of lipids (saturated long-chain alcohols) cannot be excluded a *priori*. Other compound classes, such as peptides, have been phosphorylated by PP_i_ under conditions that may be compared to those listed in Table 3 [59]. The prebiotic availability of PP_i_ on a wet Earth is still under discussion, so a kinetically more stable and perpetuously (geochemically) replenished condensing agent may be a good alternative for the large-scale synthesis of phosphate esters under prebiotic conditions. The production of condensed polyphosphates on primordial Earth, the smallest anhydride of which is PP_i_, was first attributed to volcanic activities that volatilized reduced PP_i_ precursors at high temperatures and pressures [60]. Although P_i_ is the major source of phosphorous in Earth’s crust and marine systems today [61], the availability of P_i_ on early Earth may have been quite different.

### 4.2. Reduced Oxidation State Phosphorous Compounds

More recent proposals suggest that an extraterrestrial origin of inorganic phosphorus that may have acted at different oxidation states as prebiotic phosphorylation agents producing P-reduced precursors of biochemically relevant phosphorylated building blocks [62]. Schreibersite [(Fe,Ni)_3_P], is present in many iron meteorites, so a credible hypothesis is that the Neohadean-Eoarchean Late Heavy Bombardment 4.1–3.8 Gya enriched the early Earth’s crust with reduced P minerals. The oxidation state of phosphorus in schreibersite is reported to be close to −1 [63]. Pasek and co-workers first demonstrated that a schreibersite model mineral, iron phosphide (Fe_3_P), reacts with water to give hydrogen gas, ferrous peroxyhydroxides and ferric oxides, and a huge variety of highly water soluble inorganic phosphorous compounds at different P-oxidation states. Examples include H-phosphonate HPO_3_^2−^ = P_i_(III), the favored tautomer of mono-protonated phosphite, hypophosphate (P_2_O_6_^4^^−^) bearing a P(IV)-P(IV) bond, and P(V) compounds such as P_i_ and the corresponding condensed forms, pyro- and triphosphate (PP_i_ and PPP_i_). These species are non-stoichiometrically summarized by Equation (2). In the presence of ethanol or acetic acid many different organic P compounds (phosphonic acid derivatives) form and several, such as a cyclic organic diphosphonic acid anhydride and phosphonacetate, have been identified. Electron paramagnetic resonance (EPR) spectroscopy suggested that schreibersite in water undergoes a radical reaction mechanism initiated by hydrogen (H∙), hydroxyl (HO∙) and phosphite radicals (∙PO_3_^2−^), followed by a series of radical recombination and propagation steps [64,65].

Fe_3_P + water + ethanol/acetate → n H_2_ + Fe^2+^ + Fe_3_O_4_ + PO_4_^3−^ + P_2_O_7_^4^^−^ + P_3_O_10_^5^^−^ + HPO_3_^2−^ + P_2_O_6_^4^^−^ + acyclic organic P + cyclic organic P + other identified + not identified (2)

The same authors reported on the plausibly prebiotic formation of condensed PP_i_ and metaphosphate (cyclic triphosphate = cPPP_i_) promoted by a series of radical redox reactions on water soluble H-phosphinate H_2_PO_2_^−^ = P_i_(I) through P_i_(III) using hydroxyl and hydroperoxyl radicals obtained from H_2_O_2_. The reactions could be observed in a Fenton reactor or a microwave plasma generator, thus, in the presence and, respectively, absence of ferrous/ferric ions [66]. Further support to this theory emerged from geochemical and geological investigations that showed an exceptionally high abundance of “phosphite” only in deep sediments of early Archean marine carbonates, indicating the presence of large amounts of dissolved P_i_(III) in Archean oceans [67]. Kee [68] provided geologically plausible evidence that P_i_(III) could have driven condensation of orthophosphate P_i_ into PP_i_ via pyrophosphite H_2_P_2_O_5_^2−^ = PP_i_(III) intermediates and mixed condensed isohypophosphate HP_2_O_6_^3−^ = P(III)-O-P_i_(V) under mild conditions. This perspective has led to a new scenario for the phosphorylation of lipidic amphiphiles (*n*-ROH and ILs) and biomolecular building blocks, such as serine, threonine, tyrosine, and the nucleosides, through direct phosphitylation by PP_i_(III) followed by the oxidation to known phosphate esters, APs, CLs, phosphorylated amino acids and the nucleotides, respectively. For instance, Pasek and colleagues demonstrated that reacting glycerol with schreibersite produced glycerol-1-phosphate and glycerol-2-phosphate [67], and combining aqueous acetaldehyde with Fe_3_P produced a “phosphoaldol” reaction product [63]. Feng and co-workers [53] demonstrated that the synthesis of glycerol phosphite esters is possible under plausible prebiotic conditions (Table 3, entry 11). These results indicate that, on early Earth, the phosphorylation of ILs may also have occurred in the absence of any additional condensing agents, so that PP_i_(III) may have played a central role in prebiotic phosphorylation processes.

While waiting for new scientific evidence on such alternative routes, we cannot overlook the role of condensing agents like cyanamide, or related compounds, to be considered for the synthesis of ILs, CLs and APs on the early Earth. For other aspects concerning the role of phosphorylation agents under simulated hydrothermal conditions, Gull’s recent review [69] describes how P(V) and P(III) salts can interact with mineral catalysts, urea and condensing agents to drive phosphorylation of polyols.

## 5. Cyanamide as an Universal Condensing Agent

### 5.1. Geochemistry of Cyanamide (Hypothesis)

**Figure 2:**
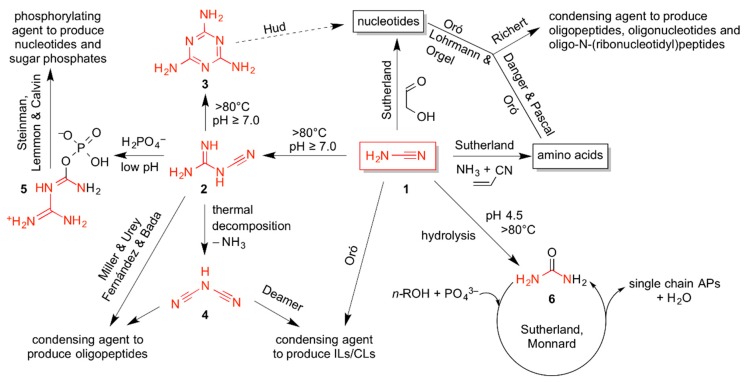
Chemical transformations of cyanamide (**1**). Cyanamide was extensively used in the 60s and 70s for the condensation of amino acids into short peptides, nucleotides, and oligonucleotides. It also acts as mono-carbon donor in the synthesis of several monomers, including amino acids and nucleotides. Under simulated hydrothermal conditions, but at different pH, cyanamide dimerizes to cyanoguanidine *alias* dicyandiamide (**2**), and eventually trimerizes to give stable melamine (**3**), or decomposes to give still reactive dicyanamide (**4**). The latter has been used for the direct condensation of amino acids into peptides and for the preparation of ILs and CLs; the former was taken responsible for the production of simple peptides from Miller’s spark discharge experiments on CH_4_, NH_3_, and H_2_O in the presence of added **1**. **2** was also used in conjunction with phosphate for the phosphorylation of nucleosides and sugars at low pH through the presumed intermediacy of *N*-[*O*-(phosphatidyl)carbamoyl]guanidine (**5**). Direct hydrolysis of **1** yields urea (**6**), another useful prebiotic coupling agent for the formation of APs when present with orthophosphate (see Figure 3).

When activated by a condensing agent, the formation of a covalent bond between a heteroatom and a carbonyl or phosphoryl group (N–C=O, O–C=O, N–P=O, O–P=O) ultimately produces a chemically bound molecule of H_2_O. The first review of condensing agents in prebiotic chemistry was edited by Hulshof and Ponnamperuma in the 1970s [70]. Many condensations of amino acids to oligopeptides and phosphorylations of amino acids, peptides, nucleosides, or sugars were recapitulated, but no synthesis of ILs, CLs and APs was described. Cyanamide (**1**) and dicyanamide (**4**) were prompted as condensing agents mainly for amide bond linkages of oligopeptides. Urea (**6**) was not mentioned. The earliest report of **1** as a possible prebiotic condensing agent for sugars, amino acids, and phosphorylation of glucose and adenosine appeared in the 1960s [71]. In that context, small amounts of **1** and its more stable dimer, cyanoguanidine (**2**) (also referred to as dicyandiamide, see Figure 2), have been shown to form upon UV irradiation of dilute aqueous HCN solutions. They are also produced by electron beam irradiation of aqueous NH_3_/CH_4_ mixtures, suggesting two possible prebiotic origins of **1** and **2** [72]. Recently, one of us [31] rationalized the origin of **1** in primordial Earth’s crust in accord with Eschenmoser’s intuitions [73]. A geochemically plausible synthesis of large amounts of **1** may have resembled its industrial synthesis, known as the Frank-Caro process. This reaction mixes calcium carbide produced from calcium oxide and carbon (Equation (3), this synthesis is industrially performed at 2200 °C according to Thomas Willson and Henri Moissan), with nitrogen at temperatures around 1000 °C to produce calcium cyanamide and carbon (Equation (4)). The standard free enthalpy of this reaction is exergonic between 850 °C and 1100 °C and only weakly temperature dependent, *e.g.*, Δ*G°*_Eq.4_ (1273 K) = −21.9 ± 1.4 kJ·mol^−1^ and Δ*S°*_Eq.4_ = −37.8 J·mol^−1^·K^−1^ [74]. Lower temperatures slow down but favor CaCN_2_ formation. Upon further cooling, residual CaC_2_ reacts with water to give acetylene and CaO (not shown), while CaCN_2_ reacts with liquid H_2_O in the presence of carbon dioxide to give calcium carbonate and aqueous cyanamide slurries (Equation (5)). CO is the isoelectronic oxygen analogue of N_2_ and H_2_NCN is the isoelectronic dinitrogen analogue of CO_2_. Thus, the overall process (Equation (6)) consists of the transformation of “burnt lime” (CaO) into “limestone” (CaCO_3_) through the oxidative release of elemental (solid) carbon as carbon monoxide gas and the reductive fixation of nitrogen gas as liquid or solid cyanamide (mp 44 °C).
  >2000 °CCaO + 3 C ⥂ CaC_2_ + CO(3)  ≈1000 °CCaC_2_ + N_2_⥂ CaCN_2_ + C(4)  ≤100 °CCaCN_2_ + H_2_O + CO_2_⥂ CaCO_3_ + H_2_N–CN(5)  Overall:CaO + 2 C + N_2_ + H_2_CO_3_ → CaCO_3_ + CO + H_2_N–CN(6)

### 5.2. Reactivity of Cyanamide and Its Homologues

Under relatively mild simulated hydrothermal conditions (65–100 °C) **1** forms **2** [75] and the stable trimer melamine (**3**) [76]. Thermal degradation of **2** gives dicyanamide (**4**), a known coupling agent for the prebiotic preparation of small peptides [70]. **4** was used to obtain low yields of CLs [48]. In Figure 2, we summarize the conversion and thermal transformation of **1** under plausibly prebiotic conditions. Some of these conditions are the same as reported for the abiotic formation of ILs and CLs [43,47,48,49]. According to Eschenmoser [73] and Sutherland [37,56], **1** may not only have acted as “fuel”, thus, as prebiotic condensing agent being hydrolyzed to urea, but also entered covalently as mono-carbonic source into pathways that lead to the abiotic formation of biotically relevant building blocks, certain amino acids and nucleotides. Hud & colleagues reported that **3** can undergo the equivalent of Watson-Crick base pairing with another simple N-heterocycle, barbituric acid. They have recently shown how these compounds are efficiently ribosylated and ribophosphorylated under simulated prebiotic conditions to produce nucleoside- and nucleotide-like compounds that spontaneously assemble in melamine-barbituric acid 3:3-hexads and grow micrometers-long non-covalent hexad-stacked supramolecular fibrous polymers [77]. This observation lends support to the hypothesis that compounds like **3** and barbituric acid could have served as precursors of ancestral information carriers, before RNA evolved [78]. Danger’s & Pascal’s recent mechanistic studies on **1** as a prebiotic condensing agent have established that, when amino acids are coupled with cyanamide to *N*-acylamino acids, thus by analogy, to the C-terminus of peptides, the coupling reaction is promoted by highly reactive C-terminal 5(4*H*)-oxazolones as intermediates [79]. Stanley Miller’s “forgotten” experimental heritage of his pioneering spark discharge experiments, yet in the presence of added cyanamide, was recently opened and analyzed by Burton, Glawin, Dworkin, Krishnamurthy & Bada, and reproduced in Fernández’s group to reveal the presence, for the first time, of a rich variety of simple peptides that ultimately formed from methane, ammonia and water [80]. Most recently, Richert and co-workers [81] tested cyanamide for the concomitant condensation of adenosine monophosphate (AMP) and glycine. After a few days of reaction, the authors detected by ^31^P-NMR spectroscopy the presence of phosphodiesters, pyrophosphate, and phosphoramidates indicating the formation of oligoadenylates, diadenosyl pyrophosphate, and *N*-(5’-*O*-adenosyl)oligoglycine phosphoramidates, respectively. Hydrolysis of **1** into **6** is achieved in a few days at 65 °C and low pH values [82]. This was confirmed by several other experimental and theoretical studies concerning the hydrolysis of **1** under different conditions (pH range, temperature and time) [83,84].

These results are summarized in Figure 2 as an update to the decades-old conjecture that many different biologically relevant molecules can be formed simultaneously by dehydration under similar, plausibly prebiotic conditions. Such reactions can occur in the absence of enzymes, mineral catalysts or even separate chemical preactivation steps, as was already demonstrated by the abiotic formation of ILs, CLs [45,49,50,51].

### 5.3. Kinetic Stability of Cyanamide and Dehydration Power of Urea

**Figure 3:**
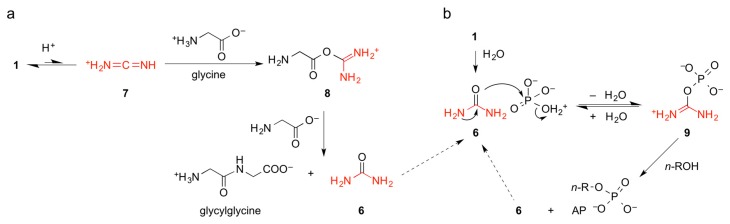
(**a**) Formation of peptide bonds using cyanamide (**1**) as coupling agent (adapted from [70], see also Scheme 2 in [79] and Scheme 1 in [80]); (**b**) Phosphorylation of long-chain alcohols (formation of APs) using urea and phosphate as condensing agent (adapted from [56]). Intermediate **9** is chemically related to intermediate **5** (*cf.* Figure 2).

As summarized by Hulsof and Ponnamperuma [70], and depicted in Figure 3a, glycylglycine is likely to be formed from glycine and **1** *via O*-glycyl isourea **8**. Further couplings to tripeptides and longer chains are additionally promoted by the rapid elimination of urea (**6**) and ring closure with the upstream carbonyl oxygen atom to give C-terminal 5(4*H*)-oxazolone intermediates [79] (not shown). The tautomerization of inert cyanamide (**1**) to reactive carbodiimide HN=C=NH is thermodynamically unfavorable and surprisingly slow at neutral or mildly basic pH values. Despite being a simple proton transfer in water, this reaction is associated with the rehybridization (orbital geometry change) of both nitrogen atoms from either sp_3_ (tetragonal for –NH_2_) or sp (digonal for ≡N) to both sp_2_ (trigonal for =NH), which gives the quantum chemical grounds for the exceptional kinetic stability of **1** in water. Since the hydrolysis of **1** can be considerably accelerated by acidification, it is reasonable to assume that its protonation to give carbodiimidium (**7**) (p*K*_a_^25 °C^ ≈ 1.0 [85]) is prerequisite for the efficient nucleophilic attack by any weak nucleophile, be it water or even the carboxylate group of an amino acid. The real chemical situation during peptide couplings promoted by **1** is probably more complicated. Parker *et al.* have shown that, at slightly elevated pH values, dimer **2** is considerably more efficient in promoting peptide bond formation than **1**, which made the authors propose a complex reaction scheme for peptide couplings involving **2** rather than **1** [80]. Much earlier, already Steinman *et al.* [71] observed that **2** was more efficient than **1** in promoting phosphorylations of adenosine or glucose using P_i_ or PP_i_. The authors postulated *N*-[*O*-(phosphatidyl)carbamoyl]guanidine (**5**) to be responsible for such dehydration reactions (Figure 2).

Thus far, it seems as if the chemical reactivity of cyanamide and its derivatives **1**–**5** in general, and their dehydrating power in particular, were suppressed or even totally quenched by their hydrolysis. The first hydrolytic product of **1** is urea (**6**) being considerably less reactive, which is chemically expected, as it would be expected from any other hydrolytic product. Partial hydrolysis of **2**, **4** and **5** gives carbamoylurea H_2_N–CO–NH–CO–NH_2_ *alias* biuret. Formally, the cyclic hydrolytic product of melamine (**3**) is cyanuric acid, and its aminolyzed ring-opened derivative is dicarbamoylurea H_2_N–CO–NH–CO–NH–CO–NH_2_ better known as triuret, which usually forms from the pyrolysis of urea. Neither urea, nor the condensed hydrolytic substances cyanuric acid, biuret or triuret are very reactive, they are not expected to possess much dehydration power. In a geochemical and prebiotic context this could indicate that, if cyanamide was present in large amounts on the early Earth (*cf.* Subsection 5.1), its chemical reactivity could only be “harvested” as long as no water, or not much of it, would be present. The kinetic stability of **1** would help maintain reactive reservoirs in water for some period of time but, where elevated temperatures (65 °C to 100 °C) prevailed, its chemical potential as dehydrating agent must have been extinguished, on geological timescales, relatively soon. The ultimate hydrolytic product of all above compounds is diammonium carbonate, thus are the volatiles carbon dioxide and ammonia.

However, already Lohrmann and Orgel reported that hydroxylapatite CaHPO_4_ was able to phosphorylate different nucleosides in the presence of **6** [82]. Powner & Sutherland [56] highlighted the important role of **6** as the first hydrolytic product of **1** in the prebiotic synthesis of pyrimidine nucleotides. Moreover, under prebiotically plausible conditions, **6** can enter a catalytic cycle in which long-chain alcohols *n*-ROH and orthophosphate P_i_ form APs [55] (Figure 2). The mechanism proposed by Sutherland (Figure 3b) means that, despite the low nucleophilicity of the urea oxygen atom and the hindered electrophilicity of the phosphorous atom (P_i_ is formally at least mono-anionic at moderately low pH), an activated form of **6**, *O*-phosphatidyl isourea (**9**) forms during evaporation at elevated temperatures (100 °C). Highly concentrated *n*-ROH is then being phosphorylated to give APs that assemble into vesicles when rehydrated at lower temperatures [56].

### 5.4. Geochemistry of Urea and Phosphate (Hypothesis)

This discovery may be a significant factor in prebiotic scenarios where slurries of concentrated **1** would be produced when CO_2_-saturated liquid water wets calcium cyanamide reservoirs (Equation (5)), which had formed at much higher temperatures in the absence of liquid water when hot calcium carbide was exposed to a nitrogen atmosphere (Equation (4)). At first, when the temperatures were close to the boiling point of water and periodic wet-dry cycles dominated, these putative CaCN_2_ reservoirs would become limestone (calcite and aragonite, CaCO_3_) and separate from the cyanamide liquids. Upon the arrival of increasing amounts of CO_2_-containing water, and in the absence of phosphate, slurries of concentrated **1** are expected to slowly hydrolyze to **6** upon dilution, followed by an ensuing dry period that evaporated volatiles again. This abiotic pathway to **6** is independent of formamide or cyanate formation [86]. A reasonably rapid drying would not let all **6** hydrolyze to volatile diammonium carbonate, so patches of crystalline urea would remain on the dried limestone surface. Time would make sure that such places would eventually hydrolyze all **6** and evaporate all nitrogen atoms as ammonia—with the notable exception of those surroundings where phosphate (or phosphite?) deposits were present. P_i_ and **6** could transiently form **9** at places where pH drops would have occurred. The chemical reactivity of **9**, as shown in Figure 2 and Figure 3b, meant that even though **1** had hydrolyzed to **6**, its reactive potential as a dehydrating agent persisted, if not unfolded, in the presence of phosphate, as long as **6** would not continue to hydrolyze to diammonium carbonate.

For the chemist this is an astonishing kind of *umpolung*: Instead of taking up water and being hydrolyzed to its thermodynamic ending, urea has been turned by phosphate into an anhydride (a dehydrating agent) working as a phosphorylation catalyst, while phosphate has become a water producer. The reason is that water seems to be a better leaving group (from H_2_PO_4_^−^ + **6** to give **9**) than urea (from **9** to give back **6**), despite having equally low basicities (p*K*_a_ ≈ −1.8 for both conjugate acids), because water is volatile and urea is not, *i.e.*, only under irreversible reaction conditions in an open system. We are now wondering about how this scenario would look like when thermal elimination of ammonia from solid or liquid hot urea (mp 133 °C) could produce significant amounts of biuret and triuret, when melamine (**3**) production would efficiently compete with cyanamide hydrolysis, and when phosphate would be replaced by phosphite or pyrophosphite. In any case, plausible sites for the synthesis of biologically relevant monomers can be regions where limestone was covered with urea and phospha(i)te salts. During periods when water was about to evaporate to dryness, these zones [33] would transiently produce high energy *O*-phospha(i)tidyl compounds, such as **5** and **9** which, upon arrival of Fischer-Tropsch products (long alkyl chain alcohols, aldehydes, or acids), Butlerov products (polyols, carbohydrates), Miller-Urey products (hydroxy acids, amino acids and peptides) and Oró products (*N*-heterocycles), could transform the accumulated compounds into “filled and functional” compartments that, upon rehydration at lower temperatures, would be ready for further evolutionary steps towards living systems. The kinetic stability of cyanamide in water, being a problem on laboratory time scales (its reactivity is quite sluggish), shows the other side of its Janus face only on geological time scales and in the presence of phosphate.

## 6. Conclusion and Perspectives

### 6.1. Conclusions

Amino acids and nucleic acids spontaneously formed on primordial Earth under the influence of different geochemical factors. Cyanamide (**1**), a putative precursor of prebiotic amino acids and nucleosides [14,21,56], and central to what has been termed “cyanosulfidic proto-metabolism” [37,38], plays a key role in experiments carried out under simulated prebiotic and hydrothermal conditions. It can serve as a condensing agent for the synthesis of lipids, including mono- or diacyl glycerols (“incomplete lipids”, ILs) or phospholipids (“complete lipids”, CLs). An interesting aspect is that aqueous **1**, being at equilibrium with its rare and reactive tautomer carbodiimide and the proton-activated carbodiimidium (**7**), can serve as a universal coupling agent in different guises, because it transforms into other reactive chemical species such as the dimer 2-cyanoguanidine (**2**), also called dicyandiamide, and dicyanamide (**4**) generated from **2** by thermal elimination of ammonia. Most importantly, its phosphorylated forms include highly reactive *N*-[*O*-(phosphatidyl)carbamoyl]guanidine (**5**) and *O*-phosphatidyl isourea (**9**). Intermediate **5** forms when orthophosphate (P_i_) reacts with **2**, and intermediate **9** is generated when **1** hydrolyses to urea (**6**) in the presence of inorganic phosphate. When condensing agents **1** and **4** are mixed with phosphate, glycerol and fatty acids or aldehydes, CLs are produced. Additionally, phosphate in conjunction with **6** can phosphorylate long-chain alcohols to the corresponding alkyl phosphates (APs) under simulated hydrothermal conditions.

However, there is as yet no experimental evidence for the direct phosphorylation of ILs to give CLs, perhaps owing to the relatively low reactivity of P(V) salts. In recent years phosphite minerals have been discovered in deep marine carbonate sediments, indicating the presence of P(III) compounds in the anoxic Archean oceans. Furthermore, it has been experimentally demonstrated that iron phosphide reacts with water as an oxidizing agent. This mineral corrodes to produce hydrogen gas, Fe(II) then Fe(III), and P(I), then P(III) and P(IV) compounds, which ultimately oxidize to P(V) without the need of molecular oxygen. Because P(III) salts such as H-phosphonate P_i_(III) and pyrophosphite PP_i_(III) are water soluble and more reactive than most phosphates, it is possible that they may have played a role in phosphitylating abiotic amphiphilic substances. For instance, CLs from crude extracts of P(V)-promoted simulated hydrothermal syntheses were able to assemble in vesicular membranes upon rehydration; so did certain crude ILs and APs, albeit only under the assistance of co-surfactants, *e.g.* prebiotically plausible single-chain alcohols, amines, or acids. But no information on the assembly of phosphitylated long-chain amphiphiles is available yet, and it could be fruitful for future studies to analyze products when P(V) salts are replaced with P_i_(III) and PP_i_(III).

### 6.2. Perspectives

One of the challenges in this field is to discover plausible reaction pathways that allow the synthesis of CLs from simple polyols (glyceraldehyde or glycerol), long alkyl chains (primary alkanols or fatty acids), in the presence of a reactive phosphorous source. The reactions should best take place at the membrane surface of vesicles under conditions that minimize the disruption or leakage of membranes that have already assembled. The goal would be to allow for an earliest possible setting in of the systemic co-evolution of the compartment components assembling in vesicles together with their macromolecular contents [87,88]. An important approach for establishing an evolvable chemical system is to supply a population of vesicles with amphiphilic components that insert into the membrane of existing vesicles, leading to vesicle growth and division, thus to the growth in population size and an evolution of “shape replicating” compartments (vesicles). To achieve this, the amphiphiles that are supplied should have a critical vesicle concentration (cvc) similar or somewhat higher than that of the amphiphiles composing the vesicles. Once inserted, the added amphiphiles, if chemically different from those in the vesicles, should eventually be transformed into “first generation” amphiphiles without diffusing out of the vesicles. Otherwise, they would form a separate set of *de novo* vesicles upon chemical transformation.

This is a major hurdle in the evolutionary transition from fatty acid vesicles to phospholipid vesicles, which requires esterification of fatty acids with, for example, phosphoglycerol. Fatty acids need 10^5^-fold higher minimal concentrations to form vesicles than phospholipids, and the average residing time of fatty acids in membranes is much shorter than that of phospholipids. As a result, any chemical reaction involving fatty acids would take place outside the vesicles, thereby interrupting the evolution of the parent vesicles’ contents. It follows that the cvc difference between the supplied amphiphiles and that of the amphiphilic components of vesicles containing macro-molecular contents needs to be minimized.

**Figure 4:**
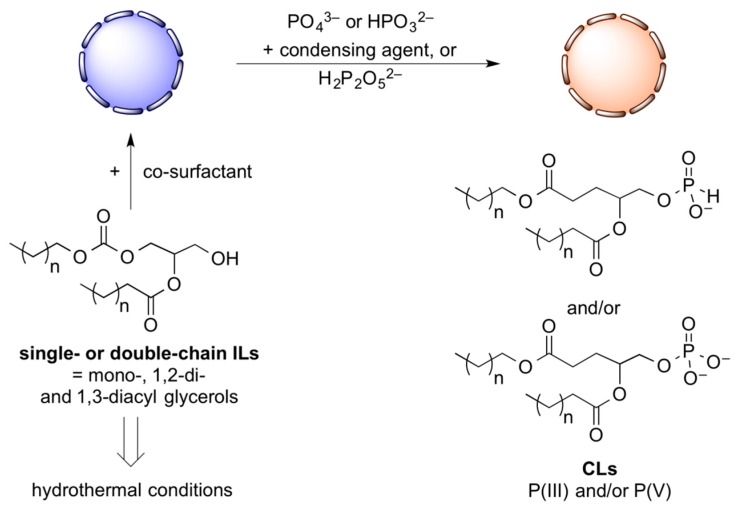
Proposed model for the direct phosphorylation/phosphitylation of ILs to CLs on vesicular membranes. In the prebiotic environment ILs can be synthesized under simulated hydrothermal conditions. In the presence of prebiotically plausible co-surfactants, such molecules can assemble into supramolecular vesicular structures (blue vesicles). Once formed, the ILs present on the surface of the vesicles are phosphorylated/phosphitylated by P(III) and/or P(V) salts in the presence of a condensing agent.

In Figure 4, we depict possible future experiments on the direct transformation of ILs (blue/grey) into CLs by direct phosphorylation or phosphitylation on the membrane surface of preformed vesicles. Mixtures of APs and co-surfactants can be fed with ILs in the presence of phosphate/phosphite sources and coupling agents like cyanamide, urea, or pyrophosphite PP_i_(III). ILs, such as mono- or 1,2-diacyl glycerols that can form boundary structures, are supplied with mixtures of a phosphorous source and condensing agent under simulated hydrothermal/prebiotic conditions. Such experiments can include encapsulated peptides and nucleic acids that have the potential to undergo co-evolution. The goal of experiments on evolvable systems [31] will be to discover primitive versions of amphiphilic vesicles containing macromolecules that can undergo evolution into more complex systems of protocells as a next step toward the first forms of life.

## Figures and Tables

**Table 1:** Phospholipid polar headgroups obtained under plausibly prebiotic reaction conditions.

Complete Lipids (CLs)		
R^3^ in Figure 1	Acronym	Head Name
H or neg. charge	PA	phosphatidic acid/phosphatidylate
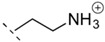	PE	phosphoethanolamine
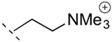	PC	phosphatidyl choline
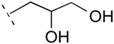	PG	phosphatidyl glycerol (glycerophosphate)
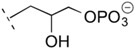	PGP	phosphatidyl glycerophosphate
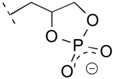	cPGP	cyclic phosphatidyl glycerophosphate
Any sugars	P(sugar)	phosphatidyl glycosides

**Table 2:** Formation of ILs under different plausibly prebiotic conditions.

Entry	Lipid Source	Condensing Agent/Promoter/Catalyst	Conditions	Products Types and Yields (%)	Analysis	Ref.
1	C_10_-C_16_ *n*-alkanes	1-naphthol	photochemical oxidation, 4 h, r.t	C_10_-C_16_ *n*-alcohols (90%)	IR; NMR and GC-MS	[39]
2	CH_4_/NH_3_/H_2_O	-	condensation 24 h, 60 °C	keto groups –CH_2_-C=O– (10%–13%)		[40]
3	^13^C oxalic or formic acid	-	Fischer-Tropsch type, 2–3 days, 175 °C	C_2_-C_35_ *n*-alcohols, *n*-aldehydes, *n*-ketones, and *n*-acids	GC-MS	[41]
4	Siderite (FeC_2_O_4_^.^2H_2_O)	-	Fischer-Tropsch type, 3–4 days, 330 °C	C_19_-C_23_ *n*-alkanes containing oxygen (“C_n_H_2n-6_O“)		[42]
5	^14^C-glycerol, ammonium palmitate	cyanamide/imidazole	condensation, 16 h, 60–100 °C	mono-; 1,2-di-; 1,3-tri and tri palmitoyl glycerol (5%–57%)	TLC; enzymatic reactions	[45]
6	C_7_-C_11_ *n*-alkanoic acids	oxalic acid	simulated hydrothermal conditions, 72 h, 150–300 °C	monoacyl-; 1,2-di- and 1,3-diacylglycerols isomers (5.9%–59.9%)	GC-MS	[46,47]

**Table 3:** Formation of CLs and phosphate/phosphite esters under different plausible prebiotic conditions.

Entry	Lipid/Alcohol Source and Phosphate/Phosphite Source (a–d)	Condensing Agent/Catalyst	Conditions	Products Types and Yields (%)	Analysis	Ref.
7	^14^C-glycerol, C_12_ fatty acids or aldehydes (**a**)	dicyanamide or silica/kaolinite;	condensation, 12 h, 65 °C	PA, PG, PGP (0.015%–0.2%)	TLC	[48]
8	^14^C-*sn*-glycero-1-phosphate, ammonium palmitate	cyanamide/imidazole	condensation, 8 h, 60–100 °C	MPGP, DPGP and cMPGP (45% of total conversion)	TLC, enzymatic characterization	[49]
9	dl-glyceraldehyde (**b**)	cyanamide/urea	reduction, 16 h, 85 °C	glycerol-phosphate (30%)	GC-MS	[50]
10	choline chloride (**a**)	cyanamide/acid traces	condensation, 7 h, 25–100 °C	PC (15%, at 80 ° C)	TLC, enzymatic characterization	[51]
11	glycerol, ethanolamine (**a**, **c**)	choline chloride:urea 2:1	condensation, 7 days, 65 °C	phosphate/phosphite esters (90%–98%)	UPLC-MS-MS ^31^P-NMR	[52]
12	glycerol, ethanolamine (**d**)	zeolite, andradite, quarz, hematite, perlite, kaolinite	3–4 days, 100–200 °C	phosphate esters (0.02%–0.98%)	LC-MS	[53]
13	C_10_ *n*-alcohols (**b**)	urea	condensation, 48 h, 100 °C	corresponding C_10_ monoalkyl phosphate (AP) and dialkyl pyrophosphate (AP)_2_	MS	[55,56]
14	dodecene (dodecyl epoxide), dodecyl phosphate	-	epoxide coupling, 24 h, r.t.	corresponding C_12_ dialkyl phosphate A_2_P	NMR	[57]

Sources of phosphate/phosphite: **a** = Na_2_HPO_4_; **b** = NH_4_(H_2_PO_4_); **c** = H_3_PO_3_; **d** = H_3_PO_4_.
